# Experimental investigation into the mechanism of secondary oxidation of coal under hot air flow erosion

**DOI:** 10.1039/d5ra02948a

**Published:** 2025-06-19

**Authors:** Peitao Zhu, Ziwen Dong, Zhenya Zhang, Song Kong, Minyang Shen, Yaxian Yu, Haojie Zhang

**Affiliations:** a School of Civil and Transportation Engineering, Ningbo University of Technology Ningbo Zhejiang 315211 China; b Zhejiang Institute of Tianjin University Ningbo Zhejiang 315211 China; c School of Safety Engineering, Ningbo University of Technology Ningbo Zhejiang 315211 China dzw2ywh2djm@nbut.edu.cn 1316859454@qq.com

## Abstract

The residual coal in the goaf of high-temperature mines is highly susceptible to oxidation and spontaneous combustion (SC) due to the erosive effects of hot airflow. Moreover, the risk of secondary oxidation may be significantly elevated. To elucidate this issue, a temperature-programmed experiment device on secondary oxidation was conducted to investigate the characteristics of coal's secondary oxidation under the influence of hot air flow with varying flow rates and temperatures. The analysis focused on O_2_ consumption rate, heat release intensity (*q*), the generation rate of carbon-oxygen gas, and the apparent activation energy (*E*_a_) during the secondary oxidation process. The results indicate that during the low-temperature oxidation (LTO) process, an increase in air flow rate leads to a higher O_2_ consumption rate and *q* for both raw coal (*R*_C_) and coal samples treated with 35 °C hot air flow (*T*_35_). Additionally, the *E*_a_ in the surface oxidation stage is lower, which implies a greater risk of coal spontaneous combustion (CSC). The temperature of the hot air flow showed a negative correlation with the rate of CO gas generation, and treatment with a hot air flow at 35 °C will promote the generation of CO_2_ gas during the later stages of oxidation. Compared with the *T*_35_ coal sample, the coal sample treated at 65 °C (*T*_65_) exhibits greater sensitivity to variations in air flow rate. Under an airflow volume of 200 mL min^−1^, the erosion of hot airflow at various temperatures can effectively reduce the risk of CSC. However, under lower airflow conditions (50–150 mL min^−1^), the *T*_35_ coal sample exhibit the highest risk of SC during the later stages of LTO. Under all airflow conditions, treatment with a hot airflow temperature of 65 °C inhibits the CSC in secondary oxidation. This investigation provides a theoretical foundation for further investigation into the mechanisms of CSC.

## Abstract 2

A temperature-programmed experiment device on secondary oxidation was conducted to investigate the characteristics of coal's secondary oxidation under the influence of hot air flow with varying flow rates and temperatures.

## Graphical abstract

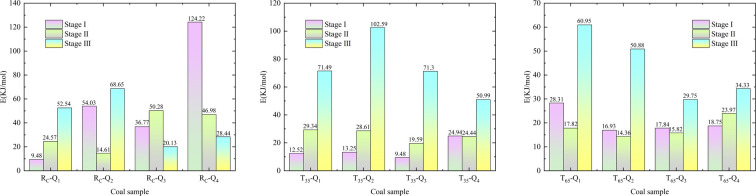

## 1. Introduction

Mine fires resulting from the CSC have consistently been one of the most significant safety hazards in coal mining operations.^1^ In China, approximately 90% of coal mine fires are attributed to the CSC.^2–4^ In recent years, as the consumption and depletion of near-surface coal resources have increased, coal production and mining activities have progressively transitioned to deeper coal seams, advancing at an annual rate of 10 to 25 m into greater depths.^5,6^ Increased mining depth results in alterations to the ambient temperature and heat storage conditions surrounding the coal, thereby increasing the risk of CSC. Studies have demonstrated that the surrounding rock temperature at a depth of 1000 m in deep mines can reach 35–45 °C. The mining depth of coal mines in China has progressed to approximately 1000–2000 m, the average temperature of a mine at a depth of 900 m in a specific region of Germany has reached 41 °C, while the geothermal temperature of a gold mine at a depth of 3000 m in India exceeds 60 °C.^7–9^ An increase in the environmental temperature of the coal seam can alter the SC propensity of coal, thereby increasing the complexity of preventing coal fires and exacerbating the risk of SC incidents.^10^

To prevent the CSC under the effect of high-temperature environments in deep mines, numerous scholars have investigated the SC characteristics of coal within deep thermal environments. Wang *et al.*^11^ conducted a comprehensive analysis of the SC characteristics of coal at varying burial depths and original rock temperatures. The results indicated that as the depth of the coal seam increases, the concentration of oxygen-containing functional groups within the coal structure also rises, thereby intensifying the propensity for SC. Niu *et al.*^12,13^ investigated the oxidation properties and heat release characteristics of coal under a deep thermal environment. They discovered that a high-temperature environment led to an increase in the number of active structures within coal, thereby enhancing its oxidation activity and thermal behavior tendencies. Zhang *et al.*^9^ used synchronous thermal analysis experiments to investigate the impact of high geothermal environments on water-immersed coal. The results demonstrated that high geothermal conditions substantially enhanced the number of reactive groups in water-immersed coal. Jia *et al.*^14^ treated coal samples at different constant temperatures to simulate the high-temperature environment in deep mines. They found that the products of gases such as CO and C_2_H_4_ during the oxidation process increased with the increase of the pretreatment temperature. The high temperature mine environment can significantly enhance the activity of functional substances in coal and increase the risk of CSC. Yu *et al.*^15^ investigated the heat release characteristics of residual coal under higher initial temperature conditions in deep mines. Research showed that an increase in the deep thermal action temperature could lower the characteristic temperature of coal, thereby enhancing its combustion performance and heat release capacity. Bu *et al.*^16^ investigated the oxidation characteristics of coal in a high-temperature environment using temperature-programmed experiment device and discovered that the concentration of carbon–oxygen gases in coal and the intensity of heat release were positively correlated with the ambient temperature.

As production activities in the mining area continue to expand downward, the intensity of air leakage (AL) with unknown patterns in the goaf will also vary in response to changes in the mining area's production activities. The erosive effect of air flow further complicates the issue of CSC.^17^ Hu *et al.*^18^ developed a mathematical model to simulate CSC in goaf areas, analyzing the impact of air flow intensity on the process. The findings indicated that increased AL results in the migration of the oxidation zone toward deeper sections of the mining area, accompanied by a rise in local O_2_ concentrations. Li *et al.*^19^ conducted further research on the impact of air flow rate on the risk of SC in coal seam groups and discovered that during the later stage of oxidation, a high air flow rate could most significantly elevate the risk of CSC. Lei *et al.*^20^ modified the external air supply conditions and investigated the characteristics of CSC using the temperature-programmed experiment device. They discovered that as the air supply volume increased, the peak corresponding to the rate of change in the O_2_ volume fraction exhibited a hysteresis effect. Liu *et al.*^21^ employed a custom-developed system to investigate reciprocating AL and discovered that, in comparison to constant airflow, reciprocating air flow can enhance the oxidation of coal and simultaneously lead to a higher production of CO gas. Pan *et al.*^22^ investigated the oxidation characteristics of coal under varying AL conditions and discovered a negative correlation between the AL intensity and its *q*. Jia *et al.*^23^ investigated the impact of variations in oxidation temperature and airflow conditions on the SC characteristics of coal using the temperature-programmed experiment device. They discovered that changes in air flow primarily influence the activation temperature and the maximum weight loss temperature of the coal sample.

To summarize, scholars have lacked research on the influence of hot air flow erosion on the SC characteristics of coal in the goaf of high-temperature mines. Moreover, compared with the primary oxidized coal samples, the SC behavior of the secondary oxidized coal samples exhibits greater complexity, making prevention and control more challenging. Therefore, it is very necessary to investigate the secondary oxidation pattern of coal samples under the erosive conditions of hot air flow. In this study, the SC characteristics of secondary-oxidized coal under varying conditions of air flow rates and hot air flow temperatures were systematically analyzed using a temperature-programmed experiment device. The oxidation kinetics of the coal samples are investigated from the perspectives of O_2_ consumption rate, carbon–oxygen gas evolution rate, and *E*_a_. By comparing the *E*_a_ of coal samples treated under different conditions during various oxidation stages, the SC behaviors of secondary-oxidized coal were comprehensively summarized. These findings provide significant guidance for understanding the phenomenon of secondary oxidation and SC of residual coal in goafs of high-temperature mines, particularly when subjected to hot air flows with differing flow rates and temperatures.

## 2. Experimental methods and procedures

### 2.1 Coal samples preparation

The coal samples utilized in this experiment were sourced exclusively from the lignite produced by Zuoyun Donggucheng Coal Industry Co., Ltd, a subsidiary of Shanxi Coal Import and Export Group. These samples were sealed in airtight bags for storage and subsequently transported to the laboratory. The liquid press device is utilized to apply a pressure of 25 MPa to the large coal sample for crushing, reducing its particle size to below 10 mm. After screening 300 g of coal samples, the samples were homogeneously mixed using the quartering method and subsequently transferred into quartz glass tubes equipped with one-way gas valves. These quartz glass tubes were then immersed in oil baths maintained at temperatures of 35 °C and 65 °C, respectively. Ventilate the interior of the glass tube and maintain it at a constant temperature for a period of 30 days. Meanwhile, a control group consisting of quartz glass tubes filled with an identical quantity of coal samples was reserved and stored at normal temperature for 30 days without being placed in the oil bath. Throughout the experiment, it was ensured that the quartz glass tubes were fully submerged in the oil and that the gas valves remained free from contact with the oil. The industrial analysis results of the experimental coal samples are presented in Table 1. The experimental treatment conditions and the nomenclature of the coal samples are presented in Table 2.

**Table 1:** Industrial analysis results of coal samples

*M* (ad)/%	*A* (ad)/%	*V* (ad)/%	FC (ad)/%
12.18	14.07	34.32	39.45

**Table 2:** Experimental treatment conditions and nomenclature for coal samples

Variable indicators	Variable indicators values	Notation
Temperature (°C)	Not oil bath treated	*R*_C_
	35	*T*_35_
	65	*T*_65_
Air flow (mL min^−1^)	200	*Q*_1_
	150	*Q*_2_
	100	*Q*_3_
	50	*Q*_4_

### 2.2 Experimental device and procedures

**Fig. 1:**
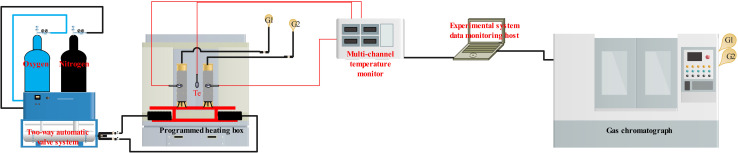
The experimental apparatus diagram.

The adopted device comprises a temperature-programmed experiment device and a chromatographic analysis system (GC-6900). Specifically, the temperature-programmed experiment device includes an electric heating blast drying oven, an FD-HQ02 dynamic gas distribution system (provided by Suzhou You Experimental Equipment Company), and a multi-channel temperature tester. The gas analyzed by the gas chromatograph is subsequently introduced into the chromatographic analysis system for detailed analysis of the gas components at the outlet. The experimental apparatus is depicted in Fig. 1.

**Fig. 2:**
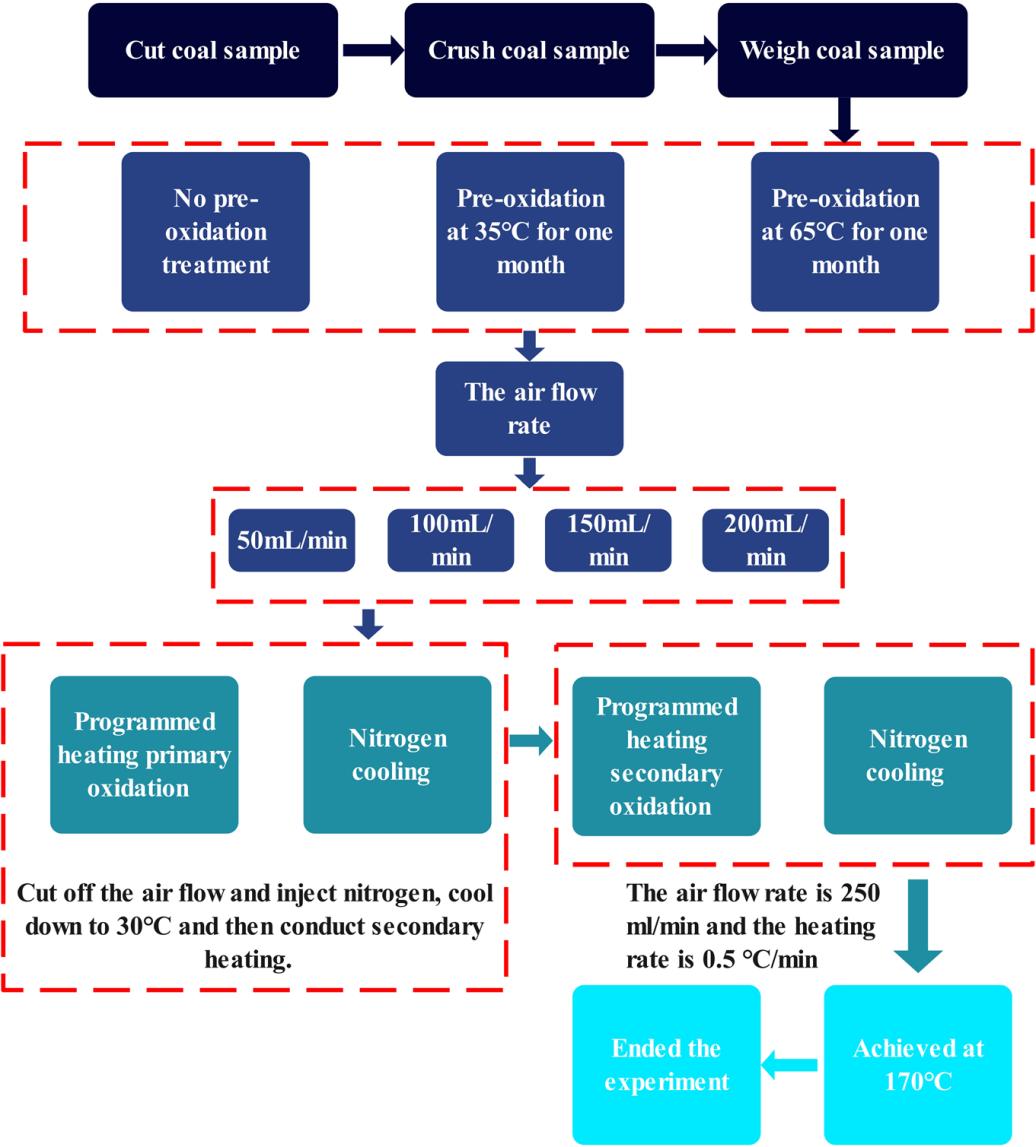
The concise flowchart of the experiment.

The coal sample tank utilized in this experiment is a copper vessel with an inner diameter of *ϕ* = 39 mm, an outer diameter of *ϕ* = 42 mm, and a height of *H* = 270 mm. The coal sample is loaded to a height of *h* = 240 mm. Use a K-type thermocouple to measure and record the central temperature of the coal sample, which is defined as the coal temperature. The copper intake pipe, with an inner diameter of *ϕ* = 3 mm, an outer diameter of *ϕ* = 4 mm, and a total length of *L* = 50 m, should be coiled and placed at the bottom of the oven to ensure that the airflow is uniformly and efficiently heated. Connect the coal sample tank to the gas supply system, configure four distinct experimental air flow rates, and perform preliminary low-temperature oxidation under room temperature conditions with a heating rate of 0.5 °C min^−1^. Cease heating when the coal temperature reaches 170 °C, subsequently replacing the air supply with nitrogen for cooling the coal sample. Set the nitrogen flow rate at 250 mL min^−1^. When the coal temperature decreases to normal temperature, restore the air supply, and re-initiate the temperature-programmed experiment device to perform the secondary oxidation of the coal sample. The procedures for the secondary oxidation are identical to those of the primary oxidation. Upon completion of the experiment, introduce nitrogen to cool the temperature-programmed experiment device, followed by opening the door of the electric thermostatic drying oven. During the secondary oxidation process, collect the gas generated at the outlet of the coal sample pot at intervals of every 10 °C. Subsequently, analyze the collected gas using a gas chromatograph. The concise flowchart of the experimental procedure is presented in Fig. 2.

## 3. Results and analysis

### 3.1 Oxidation kinetics analysis

#### 3.1.1 O_2_ consumption rate analysis

The magnitude of the O_2_ consumption rate in the coal–oxygen complex reaction serves as an indicator of the intensity of coal oxidation. Consequently, the O_2_ consumption rate is also an important indicator to judge the risk of CSC.^24^ The calculation method for the O_2_ consumption rate is presented in eqn (1):1
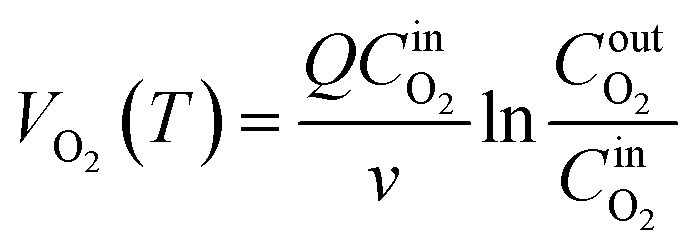
where *V*_O_2__(*T*) denotes the O_2_ consumption rate, mol cm^−3^ s^−1^; *Q* represents the air flow rate, cm^3^ s^−1^; 
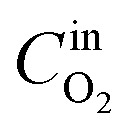
 denotes the actual O_2_ concentration at the air inlet, mol cm^−3^; *v* represents the volume of the coal sample, cm^3^; 
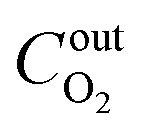
 denotes the actual O_2_ concentration at the air outlet, mol cm^−3^.

**Fig. 3:**
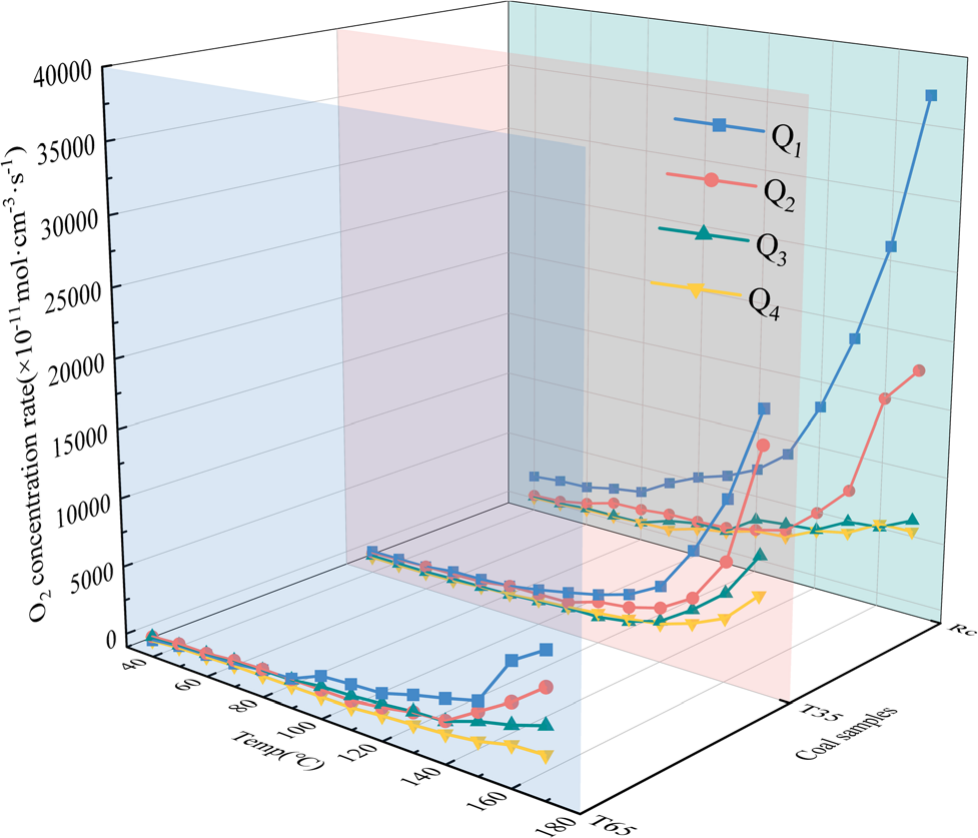
Three-dimensional curve of the O_2_ consumption rate.

The secondary oxidation O_2_ consumption rate of coal samples under the influence of various air flow rates and hot air flow temperatures is shown in Fig. 3. It can be observed from Fig. 3 that under varying air flow rate conditions, the O_2_ consumption rates of the three coal samples (*R*_C_, *T*_35_, and *T*_65_) are relatively stable in the early stage and exhibited a rapid increase in the later stage. This phenomenon can be attributed to the fact that during the later phase of the LTO process, as the coal temperature increases, the primary reactions involve chemical interactions and adsorption with O_2_, releasing substantial amounts of heat and thereby causing a marked rise in the O_2_ consumption rate. Under the *Q*_1_ air volume condition, the O_2_ consumption rate of the *R*_C_ coal sample during the entire LTO stage is substantially higher than that under other air volume conditions. However, under the *Q*_2_ air volume condition, the O_2_ consumption rate of the *R*_C_ coal sample becomes significantly higher than that under the *Q*_3_ and *Q*_4_ air volume conditions only after the coal temperature increases to 140 °C. Due to the relatively low air flow rates in *Q*_3_ and *Q*_4_, the difference in the O_2_ consumption rate of the *R*_C_ coal sample is not significant. During the later stage of the oxidation process, the O_2_ consumption rate under *Q*_3_ conditions is slightly higher compared to that under *Q*_4_ airflow conditions. Overall, as the air flow rate decreases from *Q*_1_ to *Q*_4_, the O_2_ consumption rate of the *R*_C_ coal sample exhibits an insignificant decline during the early oxidation stage. At a temperature of 170 °C, the O_2_ consumption rate of the coal sample under the *Q*_1_ air flow condition increased by 112.95%, 496.72%, and 592.88% respectively compared to those under the *Q*_2_, *Q*_3_, and *Q*_4_ air flow conditions. This indicates that throughout the oxidation process, a higher air flow rate corresponds to a greater O_2_ consumption rate for the *R*_C_ coal sample. The O_2_ consumption rate of the *T*_35_ coal sample is roughly comparable to that of the *R*_C_ coal sample. However, under the erosive influence of low air flow rates (*Q*_3_ and *Q*_4_) during the later stages of the experiment, a noticeable difference in the O_2_ consumption rate of the coal sample becomes apparent. In contrast, the trend observed for the *T*_65_ coal sample during the later oxidation stage aligns with that of the *R*_C_ coal sample, where an increase in air volume corresponds to an increase in O_2_ consumption rate. During the initial low-temperature stage, the O_2_ consumption rate decreases in the order of *Q*_2_, *Q*_3_, *Q*_1_, and *Q*_4_ under varying air flow conditions. Under identical air volume conditions, the O_2_ consumption rate trends of the *T*_35_ and *T*_65_ coal samples differ across various oxidation stages. This indicates that treatment of coal samples with hot air flows at different temperatures significantly impacts their behavior, and the coal sample becomes more sensitive to variations in air flow rates after exposure to a 65 °C high-temperature air flow.

Throughout the entire LTO process of the coal samples, the O_2_ consumption rates of the *T*_35_ and *T*_65_ coal samples under the *Q*_1_ air volume condition are significantly lower than those of the *R*_C_ coal sample. Furthermore, the O_2_ consumption rate of the *T*_35_ coal sample does not become significantly higher than that of the *T*_65_ coal sample until the temperature reaches 140 °C. These findings suggest that compared with the *R*_C_ coal sample, treatment with hot air at different temperatures inhibits the O_2_ consumption rate of the coal samples under the *Q*_1_ air volume condition. Under the airflow conditions of *Q*_2_, *Q*_3_, and *Q*_4_, the oxygen consumption rate of the *T*_35_ coal sample is consistently higher than that of the *R*_C_ coal sample before approximately 60 °C. Likewise, under these three airflow conditions, when the temperatures are below 60 °C, 90 °C, and 90 °C respectively, the oxygen consumption rate of the *T*_65_ coal sample exceeds that of the *R*_C_ coal sample. These findings suggest that under these three air volume conditions, exposure to hot air flow at varying temperatures can enhance the coal–oxygen complex reaction during the low-temperature stage of the coal samples. Compared with the *T*_35_ coal sample, under the *Q*_2_ air volume condition, the *T*_65_ coal sample exhibits a lower O_2_ consumption rate when the temperature is below 70 °C, indicating a slower oxidation reaction. Under the *Q*_3_ and *Q*_4_ air volume conditions, the O_2_ consumption rate of the *T*_65_ coal sample becomes higher when the temperatures are below 140 °C and 80 °C, respectively. In the latter phase of the LTO stage, the O_2_ consumption rate of the coal samples under the *Q*_2_ air volume condition decreases in the following order: *R*_C_, *T*_35_, and *T*_65_. Under the *Q*_3_ and *Q*_4_ air volume conditions, the O_2_ consumption rate of the *T*_35_ coal sample surpasses that of the *R*_C_ coal sample at 160 °C and 170 °C, respectively. Notably, throughout the experimental period, the *T*_65_ coal sample consistently demonstrates the lowest O_2_ consumption rate under all air volume conditions in the later stages. This suggests that during different stages of low-temperature coal oxidation, the impact of various air flow rates and hot air flow temperatures on the O_2_ consumption rate of coal samples differs significantly. Compared to the *R*_C_ coal sample, under the Q_2_ airflow condition, the *T*_35_ coal sample exhibits promotion in the early stage but inhibition in the later stage. In contrast, under the *Q*_3_ and *Q*_4_ airflow conditions, it plays a promoting role throughout both the early and late stages of coal oxidation. The *T*_65_ coal sample demonstrates promotion in the early stage and inhibition in the later stage under *Q*_2_–*Q*_4_ air volume conditions. Furthermore, during the initial stage of LTO, under the *Q*_3_ and *Q*_4_ airflow conditions, the promoting effect of 65 °C hot air flow on the coal sample is stronger than that of 35 °C, indicating that the coal–oxygen binding ability of the coal sample after exposure to 65 °C hot air flow is enhanced under these airflow conditions.

The aforementioned differences can be attributed to the fact that during the early stage of the LTO process, the treatment of coal with hot air flows at varying temperatures induces alterations in its internal structure. Specifically, mesopores within the coal gradually fragment into micropores and smaller pores. As the temperature increases, the development of micropores and small pores becomes more pronounced, thereby enhancing the specific surface area of the coal sample. This increase facilitates stronger physical adsorption between coal and O_2_.^17^ Consequently, under airflow conditions ranging from *Q*_2_ to *Q*_4_, the O_2_ consumption rate of the coal sample treated with hot air flow is significantly higher than that of the *R*_C_ coal sample during the initial phase. However, during the later stages of the LTO process, the *T*_65_ coal sample experiences a substantial depletion of functional groups due to exposure to high-temperature air flow. This reduction results in fewer functional groups available for reaction during the latter experimental stages. Thus, under full airflow conditions, the O_2_ consumption rate is observed to be the lowest.

#### 3.1.2 Carbon-oxygen gas production rate analysis

CO is produced during the oxidation reaction of functional groups in coal and serves as one of the most sensitive gases for assessing the oxidation state of coal.^25^ Both CO and CO_2_, as gaseous products, are critical indicator gases for evaluating CSC.^26^ The calculation methods for the generation rates of these two gases are shown in eqn (2) and (3):2
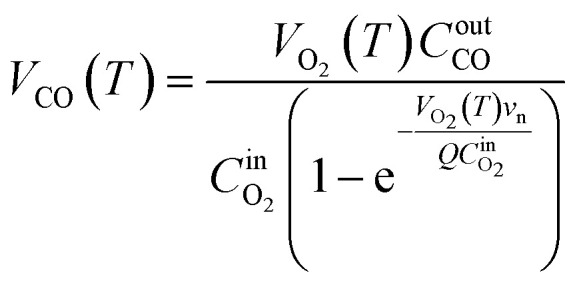
3
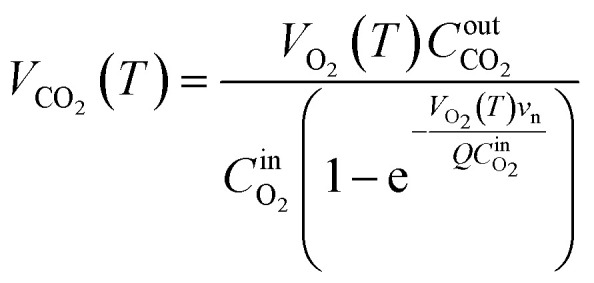
where *V*_CO_(*T*), *V*_CO_2__(*T*) represent the generation rates of CO and CO_2_ respectively, mol cm^−3^ s^−1^; *v*_n_ represents the volume of the reactor, cm^3^.

**Fig. 4:**
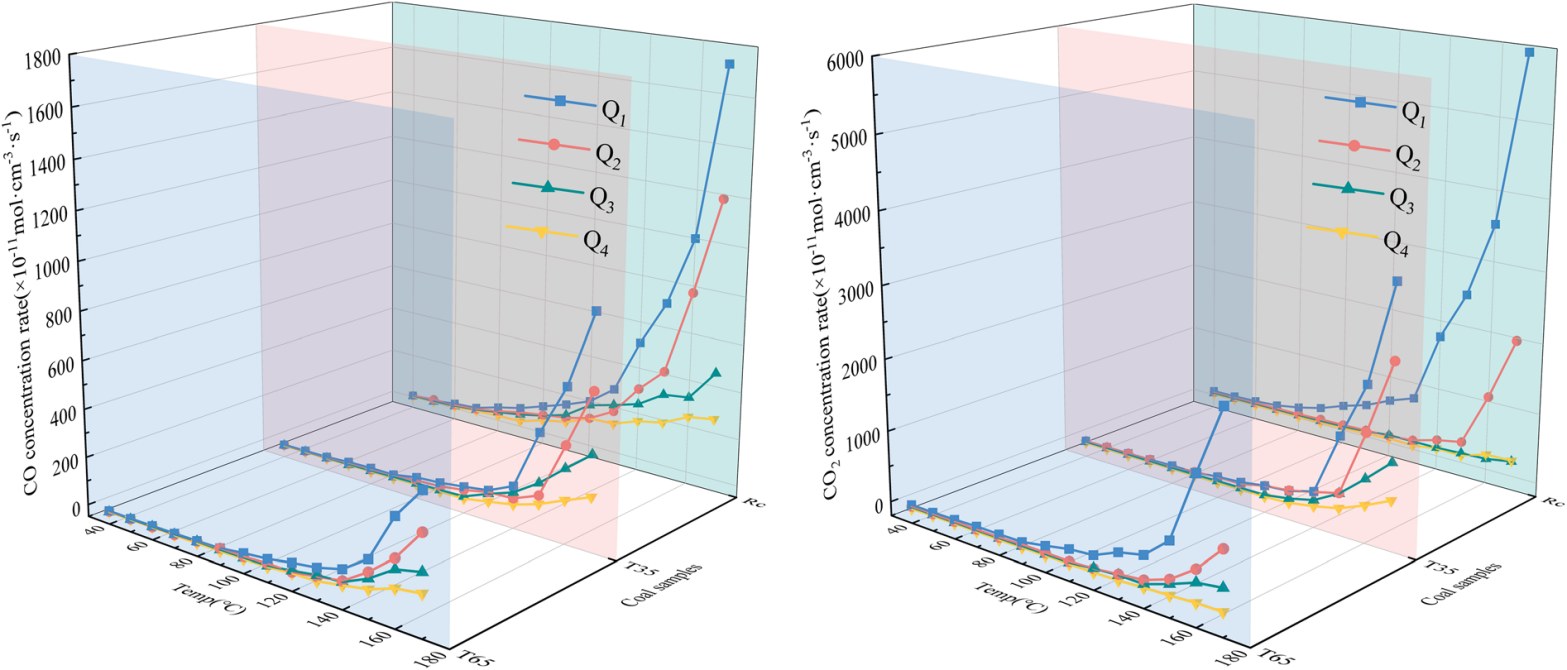
Three-dimensional curve of carbon and oxygen gas generation rate.

The CO and CO_2_ generation rates of coal sample secondary oxidation under the influence of various air flow rates and hot air flow temperatures are shown in Fig. 4. Based on the variation in the generation rate of CO gas from coal samples, two characteristic temperature points were identified for the LTO process of coal samples under the influence of hot air flow with different flow rates and temperatures. Taking the *R*_C_ coal sample as an example, the generation rate of CO gas exhibits a significant increase at 70 °C and 120 °C, which are consequently defined as the critical temperature and Xerochasy temperature, respectively. The characteristic temperature points corresponding to three coal samples under full air volume conditions are summarized in Table 3.

**Table 3:** Characteristic temperatures of coal spontaneous combustion

Coal samples	Air flow (mL min^−1^)	Characteristic temperature	
		Critical temperature (°C)	Xerochasy temperature (°C)
*R*_C_	*Q*_1_	70	120
	*Q*_2_	70	130
	*Q*_3_	70	110
	*Q*_4_	60	110
*T*_35_	*Q*_1_	90	130
	*Q*_2_	90	140
	*Q*_3_	70	120
	*Q*_4_	90	120
*T*_65_	*Q*_1_	90	140
	*Q*_2_	70	140
	*Q*_3_	70	140
	*Q*_4_	90	130

It can be observed from Fig. 4 that under varying air flow conditions, the generation rate of carbon-oxygen gas in the coal sample is consistent with the O_2_ consumption rate. Both exhibit continuous increases as temperature rises. Furthermore, as the coal temperature increases, the differences in the generation rate of carbon-oxygen gas under different air flow conditions become increasingly pronounced. Under the conditions of the same air flow rate and hot air temperature, the generation rate of CO_2_ gas in the coal sample is significantly higher than that of CO gas. This is attributed to the fact that as the coal temperature increases, the synthesis rate of CO_2_ transition complexes is considerably greater than that of CO transition complexes. Taking the *R*_C_ coal sample as an example, at 170 °C and under the *Q*_1_ air volume condition, the CO generation rate of the coal sample increased by 45.55%, 276.25%, and 563.39% respectively compared to the *Q*_2_, *Q*_3_, and *Q*_4_ air volume conditions. Similarly, the generation rate of CO_2_ increased by 201.5%, 2650.91%, and 2530.26%, respectively. These results suggest that, in general, a larger air volume leads to a higher generation rate of carbon-containing gases.

Under full air volume conditions, the CO generation rate during the later stage of the LTO process decreases in the following order: *R*_C_, *T*_35_, and *T*_65_. This indicates that higher temperatures of the hot air flow are less favorable for CO gas production. During the later oxidation stage, the CO_2_ generation rate of the *R*_C_ coal sample exceeds that of the *T*_35_ and *T*_65_ coal samples only under the *Q*_1_ condition. However, under the other three air volume conditions, when the coal temperature exceeds 140 °C, the CO_2_ generation rate of the *T*_35_ coal sample is significantly higher than that of the other two coal samples. This suggests that treatment with hot air flow at different temperatures alters the CO_2_ gas generation pattern of coal samples under *Q*_2_–*Q*_4_ air volume conditions. High-temperature treatment at 35 °C is more conducive to CO_2_ gas production during the later stages of the LTO process.

#### 3.1.3 Apparent activation energy analysis

*E*_a_ is defined as the minimum energy required to initiate and sustain the chemical reaction between coal and O_2_ under specific temperature conditions.^27^ A lower *E*_a_ indicates that less energy is needed for the coal-oxygen reaction, thereby favoring the reaction with O_2_. The data obtained from the experiment are substituted into eqn (4). Meanwhile, assuming 
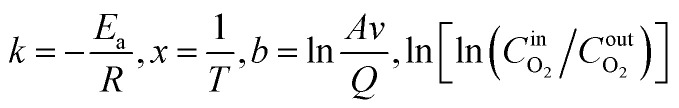
, we obtain a straight line as follows: *y* = *b* + *kx*. From the slope *k* of the straight line, the *E*_a_ of O_2_ consumption of the coal samples after different condition treatments can be calculated.4
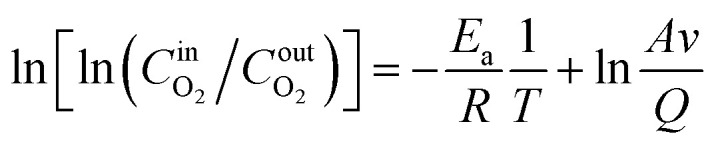
where *T* denotes thermodynamic temperature, *K*; *A* denotes the pre-exponential factor, cm^3^ s^−1^; *E*_a_ denotes the apparent activation energy, kJ mol^−1^; *R* denotes the molar gas constant, 8.314 J mol^−1^ K^−1^.

**Fig. 5:**
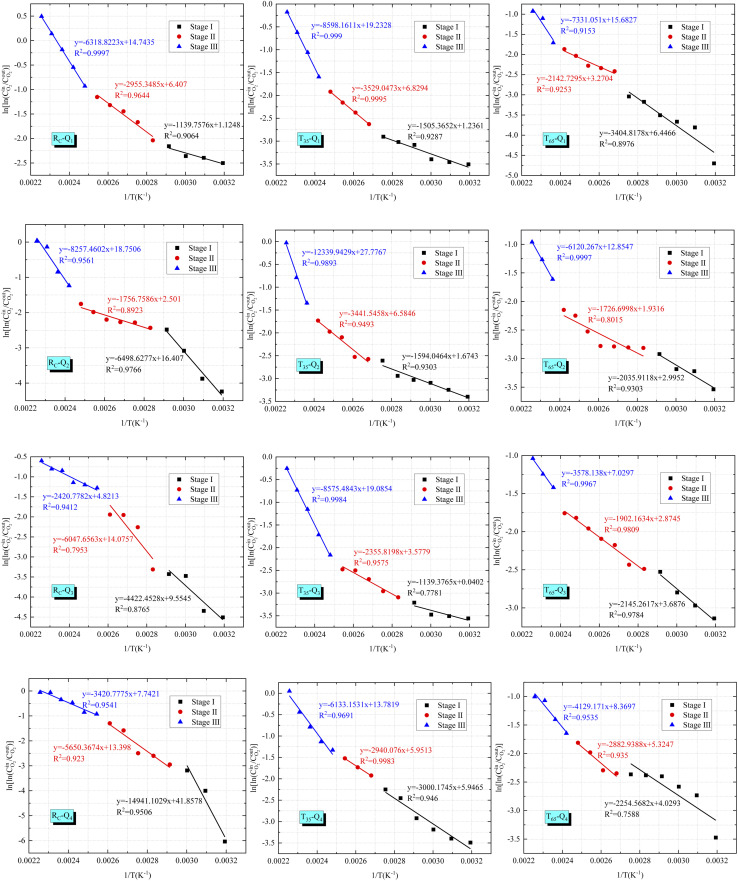
Fitted relationship between 
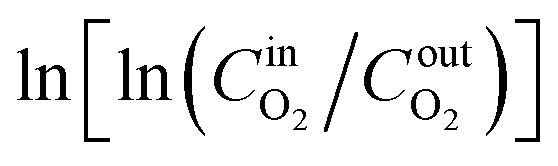
 and 1/*T* in different reaction stages.

**Fig. 6:**
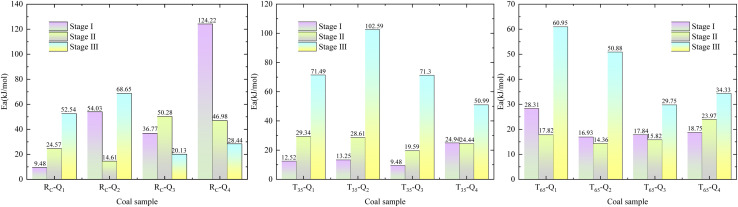
Apparent activation energy.

In order to achieve a clearer understanding of the variations in *E*_a_ across various oxidation stages during the LTO process of coal, we use the characteristic temperature points of coal samples, determined under the influence of various air flow rates and hot air flow temperatures, as the basis for the division of stages. The data were fitted using 
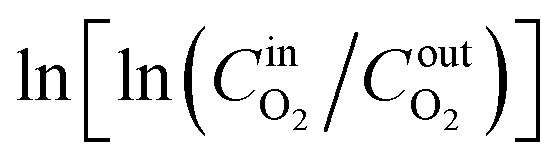
 as the ordinate and 1/*T* as the abscissa. The fitting curve and the results of the *E*_a_ are presented in Fig. 5 and 6.

Based on the Critical temperature and Xerochasy temperature, the LTO process of coal can be categorized into three distinct phases: the surface oxidation phase (Phase I), the self-oxidation phase (Phase II), and the accelerated oxidation phase (Phase III). In Phase I, as the air flow rate decreases from *Q*_1_ to *Q*_4_, the *E*_a_ of the *R*_C_ and *T*_35_ coal samples generally exhibits a gradually increasing trend. However, the *T*_65_ coal sample demonstrates a distinct phenomenon. The *E*_a_ under *Q*_1_ airflow condition is the largest. This suggests that during this stage, a higher air flow rate enhances the likelihood of oxidation reactions between O_2_ and the *R*_C_ and *T*_35_ coal samples. The anomalous behavior of the *T*_65_ coal sample may be attributed to the alteration of its internal structure caused by the excessively high temperature of the hot air flow. The *E*_a_ of the *R*_C_ coal sample under the *Q*_1_ air volume condition is reduced by 24.29% and 66.53%, respectively, compared to the *T*_35_ and *T*_65_ coal samples. Under the air volume conditions of *Q*_2_, *Q*_3_, and *Q*_4_, the *E*_a_ of *R*_C_ coal sample increased by 30.77%, 21.92%, 28.82%, 10.62%, 39.8%, and 56.27%, respectively, in comparison to *T*_35_ and *T*_65_ coal samples. In Phase III, the *E*_a_ of the *R*_C_ coal sample is generally lower than that of the *T*_35_ and *T*_65_ coal samples under identical air volume conditions. It is evident that, during the initial phase of LTO, the *R*_C_ coal sample requires less energy for the oxidation reaction with O_2_ compared to coal samples treated at two other temperatures under the *Q*_1_ air volume condition. This suggests that the coal–oxygen reaction is easier to proceed in this scenario. Furthermore, during the initial stage of oxidation, the *E*_a_ of the *T*_35_ and *T*_65_ samples is lower under *Q*_2_ to *Q*_4_ air volume conditions, indicating a reduced energy requirement for the coal–oxygen complex reaction. In the later stages of the LTO process, the *E*_a_ of the *T*_35_ and *T*_65_ coal samples increases under full air volume conditions. This phenomenon may be attributed to the consumption of a significant number of active functional groups during the LTO phase, necessitating oxygen molecules to react with more stable functional groups in the later stages of oxidation, thereby reducing the likelihood of continuous development.

#### 3.1.4 Heat release intensity analysis

**Fig. 7:**
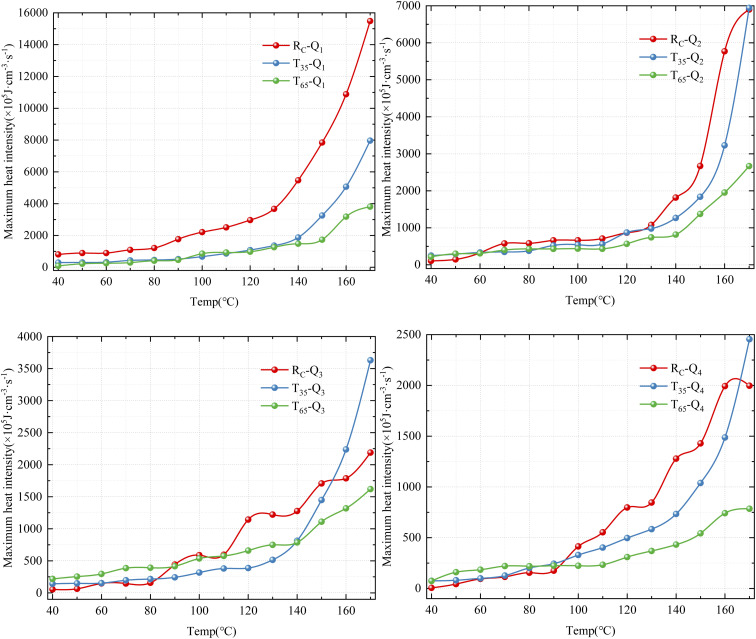
The maximum heat release intensity.

The exothermic oxidation capacity of coal serves as a critical parameter for evaluating the propensity of coal to undergo SC. When the heat released exceeds the heat dissipated, over time, the coal mass continuously accumulates heat and eventually leads to SC. Investigating the *q* of coal is essential for comprehending the mechanism of CSC.^27,28^ This paper conducts an analysis based on the calculation formula for the maximum *q* presented in eqn (5). Examining the most unfavorable factors affecting residual coal in goafs can significantly enhance the prevention and control of CSC.5
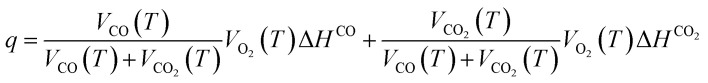
where *q* denotes the maximum heat release intensity, J cm^−3^ s^−1^; Δ*H*^CO^, Δ*H*^CO_2_^ represent the average reaction heat of CO and CO_2_, Δ*H*^CO^ = 311.9 kJ mol^−1^, Δ*H*^CO_2_^ = 446.7 kJ mol^−1^;

The maximum *q* of coal samples under the influence of various air flow rates and hot air flow temperatures is presented in Fig. 7. It can be seen from the figure that the trend of the maximum *q* during the secondary oxidation process of the coal sample is basically consistent with the O_2_ consumption rate. Under the condition of full air flow, the maximum heat release intensities of the three coal samples all increase continuously with the increase of the coal temperature, and the differences gradually become obvious. During the entire process of LTO, the larger the air volume, the greater the maximum *q* of the *R*_C_ and *T*_35_ coal samples. The *T*_65_ coal sample exhibits a similar trend in the later stages of the LTO process, but it differs significantly during the initial stage. Under medium air volume conditions (*Q*_2_ and *Q*_3_), the *q* of the coal sample is notably higher. Conversely, under conditions of both maximum and minimum air volume (*Q*_1_ and *Q*_4_), the *q* is observed to be the lowest. Under identical air volume conditions, the regularity of *q* for coal samples treated with hot airflow at varying temperatures changes, suggesting that temperature variation significantly influences the exothermic capacity during coal oxidation.

The maximum *q* of the coal samples under the *Q*_1_ air volume condition decreases in the following order: *R*_C_, *T*_35_, and *T*_65_. This indicates that the higher temperature of the hot air flow inhibits the occurrence of CSC for the *Q*_1_ airflow condition. Under air flow conditions ranging from *Q*_2_ to *Q*_4_, the maximum *q* of the *T*_35_ coal sample surpasses that of the *R*_C_ coal sample during the later stages of LTO. In contrast, the *T*_65_ coal sample consistently exhibits the lowest exothermic intensity. This suggests that, compared to the *R*_C_ coal sample, high-temperature treatment at 35 °C enhances the chemical reactivity of coal with O_2_ during the later stages of oxidation, thereby increasing the heat release associated with oxidation. However, exposure to excessively high-temperature treatment at 65 °C has an adverse effect, significantly suppressing the heat release behavior of coal during the later stages of oxidation. In the early oxidation stage, under *Q*_3_ and *Q*_4_ air volume conditions, the *T*_65_ coal sample exhibits the highest maximum *q*, followed by the *T*_35_ coal sample. This suggests that high-temperature treatment enhances heat release during the initial oxidation phase of coal samples compared to the *R*_C_ coal sample. Furthermore, the promoting effect of 65 °C high-temperature treatment on the heat release capacity of coal oxidation is more pronounced than that of 35 °C high-temperature treatment. Similarly, it can be observed that, compared to the *Q*_1_ air volume condition, *Q*_2_ to *Q*_4_ air volume conditions provide sufficient O_2_ to the *T*_35_ and *T*_65_ coal samples without disturbing their heat storage phase.

## 4. Conclusions

To investigate the characteristics of CSC during the secondary oxidation process under high-temperature deep well goaf conditions, the temperature-programmed experiment was conducted to determine the O_2_ consumption rate, carbon oxide generation rate, *q*, and *E*_a_ of coal. A comprehensive analysis is performed on the oxidation kinetic behavior of coal samples under varying conditions of air flow rates and hot air flow temperatures, leading to the following conclusions:

(1) The coal samples exhibit higher O_2_ consumption rates and *q* under conditions of increased air volume during the LTO stage. This increases the risk of CSC. Under an air flow rate of 200 mL min^−1^, the O_2_ consumption rate and *q* of coal samples treated with hot air flow at various temperatures are substantially lower than those of the *R*_C_ coal sample, and the risk of CSC is reduced. Under flow rates ranging from 50 to 150 mL min^−1^, treatment with hot air flow at varying temperatures can enhance the coal–oxygen complex reaction capability of coal samples during the initial low-temperature stage. Furthermore, the promoting effect of hot air flow treatment at 65 °C is more pronounced compared to that at 35 °C under airflow conditions of 50 to 100 mL min^−1^. Nevertheless, during the later oxidation stage, the *T*_35_ coal sample exhibit the highest risk of SC.

(2) Under full air volume conditions, there is a negative correlation between the temperature of the hot air flow and the generation rate of CO gas, meaning that higher temperatures of the hot air flow are less conducive to CO gas production. Under air flow rates ranging from 50 to 150 mL min^−1^, treatment with a 35 °C hot air flow is more favorable for CO_2_ gas production during the later stages of the LTO process.

(3) In the surface oxidation stage, an increase in the air flow rate results in a decrease in the *E*_a_ for both the *R*_C_ and *T*_35_ coal samples, thereby enhancing the likelihood of oxidation reactions with O_2_. At an air flow rate of 200 mL min^−1^ during this stage, the oxidation reaction of the *R*_C_ coal sample with O_2_ requires less energy compared to other coal samples. Conversely, at air flow rates ranging from 50 to 150 mL min^−1^, the *E*_a_ of coal samples treated with hot air flow is lower than that of the *R*_C_ coal sample, facilitating easier progression of the coal–oxygen reaction.

## Author contributions

Peitao Zhu: writing – original draft; data analysis and writing-original draft. Ziwen Dong: investigation; writing –review & editing; funding acquisition. Zhenya Zhang: criticism. Song Kong: investigation; formal analysis. Minyang Shen: methodology; supervision. Yaxian Yu: criticism; supervision. Haojie Zhang: resources; project administration.

## Conflicts of interest

The authors declare no competing financial interest.

## Data Availability

All relevant data are within the paper.
